# Translation, Adaptation, and Validation of the L’Échelle d’Interactions Infirmière-Patient-23 for the Portuguese Culture: The Multidimensional Nature of Nursing Care

**DOI:** 10.3390/ijerph182010791

**Published:** 2021-10-14

**Authors:** Paula Agostinho, Filomena Gaspar, Teresa Potra

**Affiliations:** 1 Local Health Unit of Castelo Branco, Corporate Public Entity, 6000-085 Castelo Branco, Portugal; 2 Nursing Research, Innovation and Development Centre of Lisbon (CIDNUR), Nursing School of Lisbon, 1600-190 Lisbon, Portugal; mfgaspar@esel.pt (F.G.); tsantos@esel.pt (T.P.)

**Keywords:** clinical nursing, nursing assessment, psychometric properties, validation study

## Abstract

Nursing care is based on the interaction between nurse and patient. The L’Échelle d’Interactions Infirmière-Patient-23 (EIIP-23) is used to evaluate and understand the perception of nurses about their interventions in the practice of care, to reach better health results. The present study aims to validate the questionnaire EIIP-23 to Portuguese, evaluating its psychometric properties. Methods: This is methodological research for the process of cross-cultural translation and adaptation. Results: The process of cross-cultural translation and adaptation were satisfactory. The committee of experts reached an agreement of more than 90% in the first evaluation for all the items. The internal consistency of the nurse-patient interaction scale 22-PT (NPIS-22-PT) was 0.864. Exploratory and confirmatory factor analyses were carried out in the NPIS-22-PT model, with three factors. The results show that the final factorial solution presents acceptable goodness of fit indexes and adequate convergent validity. Conclusion: The translated version produced a good quality psychometric evaluation, and can be considered a valid, trustworthy, and useful instrument to evaluate the nurse-patient interactions in Portugal. It showed acceptable reliability and validity in psychometric tests. In the context of nursing, the NPIS-22-PT is a relevant instrument.

## 1. Introduction

Studies about nursing-patient relations are scarce in literature. Although the concept of nursing care is multidimensional, involving a set of dimensions and conditions [1], it is essential to evaluate it to monitor the quality of nursing care [2]. It is a consensus that nurses, as health professionals, have an influence on the satisfaction of patients, especially in the context of a hospital [3]. Nurses spend more time with hospitalized patients than other health workers and interact more often with them. As a result, they have a significant impact on the experience of hospitalization and have the opportunity of becoming closer to patients and to know their expectations [4]. It is relevant to verify whether nurses notice attitudes and behaviors during their professional practice, making it pertinent to use valid and reliable measurement instruments [5] to improve the quality of health services [6].

The L’Échelle d’Interactions Infirmière-Patient-23 (EIIP-23), elaborated by Cossette, Côté, Pepin, Ricard & D’Aoust [7], describes the attitudes and behaviors of nurses in their professional practice [8], making it possible to evaluate them according to importance, frequency, satisfaction, and competence [9]. A fundamental reference in nursing emerges from nursing-patient interactions, since they enable attitudes and behaviors in the humanistic, relational, and clinical domains of nursing practice, being the main vehicles to promote the quality of nursing care [10]. Each interaction with the patient is an opportunity for a curative intervention and has positive effects over patient care, the progression of the disease, and the adherence to the treatment [11].

The EIIP-23 evaluates the practices of nursing care based on the deductive and inductive processes that describe the attitudes and behaviors of nursing care, corresponding to ten carative factors. From all instruments available [12], it presents a good psychometric quality and viability (Cronbach’s alpha = 0.90; χ^2^ [811,43] = 10,311.27, *p* < 0.0001; CFI = 0.98; RMSE = 0.070) [13].

The EIIP-23 was projected to evaluate, specifically, nurses, in many clinical fields (including intensive care units and hospitalization units). There are four studies involving its translation and validation into Chinese [14], Korean [15], Filipino [16], and Arabic [17].

Considering the importance of evaluating and understanding the perception of nurses about their interventions in the practice of care, in order to achieve better outcomes in health, this study aimed to translate, adapt, and validate the EIIP-23 questionnaire into Portuguese, evaluating its psychometric properties.

## 2. Materials and Methods

### 2.1. Study Design

This is methodological research for the process of cross-cultural translation and adaptation to Portuguese, which had six stages: translation, synthesis of the translations, back translation, submission to the committee of specialists, pre-test, and opinion of the original author. The internal consistency was analyzed using Cronbach’s alpha and exploratory and confirmatory factor analyses. The software used for data analysis was the The statistical software IBM-SPSS Statistics version 26.0 and AMOS (IBM Corp, Armonk, NY, USA) were used to carry out all analyses.

### 2.2. Data Collection and Procedure

The process of translation and cross-culture adaptation of EIIP-23, guided by the theoretical references from Beaton, Bombardier, Guillemin & Ferraz [18], included the following stages: I—first translation; II—synthesis of translations; III—back translation; IV—specialist committee; V—pre-test; and VI—final version of the Escala Interação Enfermeiro-Paciente (the nurse-patient interaction scale-PT) for the Portuguese culture of Portugal. 

Stage I, which is the first translation from the original French version into the Portuguese culture of Portugal, was carried out independently by two bilingual translators. One of them was fluent in French and had knowledge about the field and the goals of the study, which made it possible to achieve cultural and idiomatic equivalence (T1). The second translator worked without this knowledge (T2). After the independent translations, stage II was carried out. In it, the translators got in touch to discuss the synthesis of the versions T1 and T2, generating the first version in Portuguese.

In stage III, a back translation was made, starting with the translated versions (BT1 and BT2). Two other bilingual (French/Portuguese) translators, with no training in the field of health, translated the questionnaire back to the original language. This is a process to verify the validity and guarantee that the translated version precisely reflects the content of each item in the original version.

In stage IV, the specialist committee carried out an analysis with the participation of a professor with a PhD in linguistics, two professors with PhDs in nursing and broad knowledge about the theme being addressed, and two MS nurses, both of whom had knowledge about the methodological process of cultural adaptation. After the version was translated and synthesized, it was evaluated and compared to the original one. The main role of the specialist committee was to compare the versions, evaluating their semantic, idiomatic, cultural, and conceptual equivalence [18]. Semantic equivalence refers to evaluating the meaning of the words to preserve their original meaning; idiomatic equivalence, to formulate idioms and colloquial expressions that are equivalent in the target-language; cultural equivalence refers to routine terms and situations that are different between the cultures; and conceptual equivalence refers to the words which have cultural meaning. Thus, the pre-final version was generated. The relevance and representativity of the items were evaluated using the content validity index (CVI), which measures the agreement between evaluators. The fitness of each item was evaluated as “fit” or “not fit”, and a minimum value of 0.80, or 80%, was considered [19].

Later, in stage V, the pre-test was carried out, which was an experimental application of the questionnaire. Each nurse received the pre-final version, a guide to apply the questionnaire, and the document for recording the evaluation and potential suggestions. The objectives of the pre-test were explained, and it was made clear that its structure could not be changed (the number of questions or their order, as well as the response options). The objective was for them to analyze the way in which the questions were formulated (clarity and degree of understanding), the acceptability of the words used, as well as their cultural relevance. The time spent to respond to the questionnaire varied from five to fifteen minutes. Since there were no difficulties in understanding the statements, and no doubts regarding their form or content, the pre-final version did not need to be reformulated, thus leading to the final version.

The final stage was the submission of all translations, back translations, specialist reports, and notes to the authors of the original instrument [18]. Once the authors received this information, they could verify whether all stages were carried out correctly to validate the final version of the instrument. 

The translation and synthesis stages were carried out satisfactorily. In the back translation, the versions of translators 1 and 2 were identical in 19 statements, and the differences were found to be due to the use of synonyms. Therefore, it was found that the back translations matched the original instrument.

The original and final versions were evaluated by the committee of specialists regarding their semantic, idiomatic, cultural, and conceptual equivalences. The percentage of agreement about the items was calculated according to the CVI, and there was an agreement above 90% in all items in the first stage of the evaluation. In addition to the evaluation of the specialists about whether the terms were adequate or not, they presented a report with suggestions for change and their justifications. The specialists suggested three terms that should be changed with regard to text equivalence, among which: in “d’intervenir avec moi”, used in item 7, the specialists suggested replacing “intervir comigo” (“intervening on me”) by “na prestação de cuidados” (“when providing care”); in item 11, there were doubts about the translation of “à explorer”, which could mean “explorar” (“to explore”), and after the authors clarified that the term meant “go towards”, the item was changed to “that meet”; in item 16, “ont cherché” implies “procurar” (“to search”), but the specialists suggested “helping them”, and this term was chosen for the version for the Portuguese culture of Portugal. After the committee of specialists reached a consensus, the suggestions were analyzed by the researchers, and all recommendations regarding textual content were accepted for the creation of the pre-final version, which was later applied in the pre-test.

After these items were reviewed, a final version was reached, which was sent to the authors of the original version for approval. They approved the final version, and thus the Portuguese cultural version of the nurse-patient interaction scale-PT was created.

#### Participants

Our sample was formed by 147 nurses working in a health organization in Portugal. Mâroco (2018) recommends 5 responses for each item. Responses from 6.4/item were recorded, which is very good. The sample of the study was non-probabilistic and intentional. The instrument was applied in eight hospitalization units: gastroenterology; orthopedics; cardiology; medicine; surgery; urology; and nephrology. The criteria of inclusion considered all nurses from hospitalization services with two or more years of work experience. Application took place over two consecutive months, from October to November 2019.

### 2.3. Data Collection Instrument

The EIIP-23 is formed by 23 items and measures 4 dimensions related to clinical care. These correspond to the health problems of the patient (items 1 through 9); relational care: which emphasizes the main elements of a therapeutic relation that involves the perceptions of the patient about a particular situation, such as the relation of help, the expression of emotions, the solving of problems, and spirituality factors (items 10 through 16); humanistic care: which reflects on the interdependence of care and humanism, hope, and sensitivity (items 17 to 20); and comfort care: a relation of care that protects, reinforces, and preserves the dignity, humanity, and integrity of the patient (items 21 and 23) [9]. The items are evaluated regarding the importance nurses give to them in their provision of care, according to a 5-point Likert scale, varying from “not important at all” to “extremely important”. The items are evaluated regarding the frequency they have been performed by the nurse in the last two weeks by a 5-point Likert scale, from “almost never” to “almost always”.

In this study, the instrument for data collection distributed in the sample is formed by two parts: Part A, general characterization, in which sociodemographic and professional characterization was carried out; and Part B, nurse-patient interaction scale. 

Part A is assessed by closed questions. In Part B, the importance of the items will be evaluated using a Likert scale, “not important at all”, “of little importance”, “moderately”, “very important”, and “extremely important”, while the frequency will be evaluated as “almost never”, “sometimes”, “frequently”, “most times”, and “almost always”. The nurses were asked two questions about each item: (a) How much your provision of care to the patients is “important” for you? and (b) In the last two weeks, how “often” did you provide care to the patient? Then, each of these questions received a score from 1 (not important at all) to 5 (extremely important), and 1 (almost never) to 5 (almost always), respectively, for each of the 23 items. As the author of the instrument Cossete et al. (2006) [7], we evaluate the scale items with the average of the two dimensions, i.e., frequency and importance that vary between 1 and 5 points for the 23 items. The importance dimension refers to how important their care delivery is for nurses, measured by a 5-point Likert scale from “not at all important” to “extremely important”. Frequency refers to how often the nurse performed it in the context of their care, measured by a 5-point Likert-type scale of “almost never” to “almost always”.

### 2.4. Ethical Considerations

Before the research was carried out, the author, who provided the questionnaire in French, gave her formal consent. The project earned a favorable opinion from the Ethics Committee of the health organization No. EC201901206, for putting the study in practice.

## 3. Results

From a total of 147 nurses, 83% were female. Their mean age was 42.6 years old, with a standard deviation of 10.1 years. General care nurses represented 88.4% of the sample, 5.4% were specialist nurses and 6.1% were nurse managers. The mean time of professional experience was 18.6 years, with a standard deviation of 10.1 years. 

### 3.1. Exploratory Factor Analysis

For the exploratory factor analysis, Bartlett’s test of sphericity was significant (χ^2^ = 231; *p* < 0.001), and the KMO index was 0.779, a medium recommendation for the analysis of the main components [20].

Item 20 was not in accordance with the criteria for factor loading above 0.40, so, it was excluded [21]. The nurse-patient interaction scale 22-PT (NPIS-22-PT) found three components that explain 57.546% of the total variance. The scale, in general, includes 22 items in three components. The first component, “relational care”, has 10 items, which evaluate aspects related to the capacity of nurses in dealing with the challenges perceived in the patient, including the capitalization of the information transmitted, considering aspects such as the personalization of care, respect for privacy, for personal needs and characteristics, the promotion of wellbeing, and the readiness of care [9]. The second component, “clinical care”, has 8 items, which evaluate aspects regarding self-care. It can be associated with personal posture concerning others and the future. This attitude influences the way in which nurses perceive their health conditions, interact with their patients, and position themselves concerning the treatment decided upon. They are attitudes that influence and are influenced by the functional capacity for self-care, self-esteem, and satisfaction with life [9]. The third component, “comfort care”, includes 3 items, which report on the way nurses perceive the efficacy of the process and evaluate aspects related to interaction in the treatment with regard to the transmission of information in an understandable way, their ability to listen, to solve problems timely, to respond to their needs, and to show technical competence [9]. 

We believe that it is necessary to maintain the perspective from Cossette et al. [7], so, we kept the name of the three factors in our model, only changing the semantics or meaning in the dimension “relational care”.

### 3.2. Reliability Analysis

The NPIS-22-PT presented acceptable levels of reliability (α = 0.864) of the total score for each of the two questions placed (importance and frequency). Cronbach’s alpha values for the three components varied between dimensions. “Relational care” had α = 0.910; “clinical care”, α = 0.827; and “comfort care”, α = 0.755, all results were considered good according to Almeida [20] (Table 1).

### 3.3. Confirmatory Factor Analysis

The confirmatory factor analysis was carried out in the structure of three NPIS-22-PT factors, found in our sample in the two local health units of Portugal. Although the NPIS-22-PT items presented good factorial weight (>0.4), at first, the CFA model showed inadequate fitness (χ^2^/gl = 875.8; CFI = 0.659; GFI = 0.665; RMSEA = 0.149; MECVI = 6.763).

Furthermore, the Mahalanobis distance indicated the presence of several multivariate outliers, some of which were removed from the model, and the modification indices were analyzed.

Since the changes were not significant, this study analyzed the indices of modification of the model. The largest ones involved the correlations of mistakes between the items 6 and 7, and 8 and 9, and between the items 16 and 17. When items from the same factor have related errors, it is common to add this trajectory to the model, justifying it, from a theoretical point of view, due to the similarity of the formulation or the content of the items.

**Figure 1:**
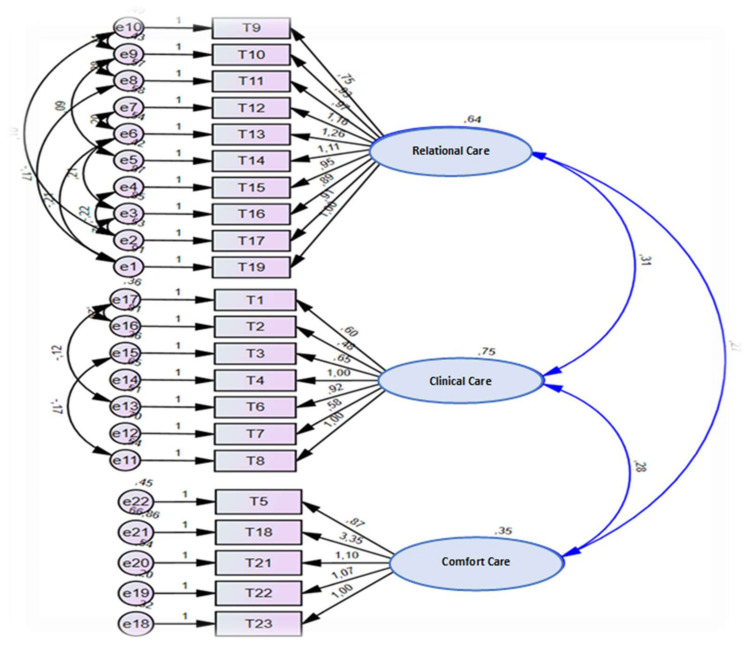
Three-factor model of the nurse-patient interaction scale (EIIP-22 PT) in the hospital care in Portugal.

Considering the revision, the model (Figure 1) presented better goodness of fit indices and a lower MECVI (5.573 versus 6.763) than the one found in the starting model.

The reliability of the construct showed that the scale had an adequate internal consistency with a high level of internal consistency, with good reproducibility in its subscales (Table 2). Considering the average variance extracted (AVE) [21] as an indicator of the convergent validity, the scale was found to be adequate in all its factors: “relational care”, “clinical care”, and “comfort care”.

The analysis of the factorial invariance of the model in both independent subgroups (test and validation) showed adequate goodness of fit indices in the final factor solution (χ^2^/df = 588.8; CFI = 0.859; GFI = 0.828; RMSEA = 0.100 (90% CI = 0.092–0.109, *p* = 0.000); SRMR = 0.0725; MECVI = 5.573).

## 4. Discussion

This study aimed to translate, adapt, and validate the L’Échelle d’Interactions Infirmière-Patient-23 (EIIP-23) questionnaire in Portugal, evaluating its psychometric properties in the hospital context of Portugal. This is the first study to validate the EIIP-23 in the country and the fifth international one. These reasons reiterate how important and relevant this study is on national and international levels.

The results provide empirical evidence that indicate an adequate psychometric performance of the NPIS-22-PT in the context of Portuguese hospitals. The model, which has three components, is conceptually consistent with the one suggested by the author of the original scale, Cossette et al., (2008) [13], based on exploratory and confirmatory analyses.

The item excluded (20) from the factorial analysis, belonged to the dimensions “Humanistic Care”, from the original version [13], and, as a result, this study generated a shorter version of the L’Échelle d’Interactions Infirmière-Patient-23 (EIIP-23).

All coefficients were significant. The coefficient of the fitness indices of the model were satisfactory and gave support to a three-factor factorial structure. Furthermore, the invariance of the three-factor model confirmed its stability. The internal consistency values found in the three-factor model of our study (α = 0.864) is lower than the values found by Cossette et al. [13] (α = 0.82), Im et al. [15] (α = 0.95), Calong et al. [16] (α = 0.94), and Jiang et al. [14] (α = 0.97); it is the same found by Atar & Aşti [22] (α = 0.79). However, the items from each component differ when compared to the studies from other countries. In the Chinese version by Jiang et al. [14] and Korean by Im et al. [15], the number of participants was higher than in our version of our study, this difference being enough to improve internal consistency. Regarding the analysis of the Portuguese version, it can be said that the measure showed good levels of internal consistency in the global dimensions and in the dimensions considered. Furthermore, it seems to be adequate in terms of content validity, considering the linguistic equivalence process developed and the conceptual agreement between the panel of evaluators.

These differences in structure, regarding both the number of items and their distribution in the subscales, occur following a confirmatory factor analysis with the aim of better establishing the psychometric properties of the instrument and reflecting existing cultural differences.

### Limitations

A limitation of this study is the lack of studies that translated and validated the EIIP-23 in other cultures, which limits the analyses of the methodological process, making it more difficult to discuss and compare results to evaluate the evolution of the level of nurse-patient interactions, thus contributing to improvements in nursing care.

## 5. Conclusions

This study made it possible to translate and adapt the L’Échelle d’Interactions Infirmière-Patient-23 for the Portuguese context, contributing towards the advancement of knowledge and evidence-based practice, as it provides an instrument that can evaluate nurse-patient interactions in the provision of care and the results of patients in Portugal. Carried out in a hospital context, with a sample of Portuguese nurses, it provided psychometric evidence and revealed an adequate three-factor structure for the NPIS-22-PT. The evidence supports the reliability and validity of the structure and is in accordance with the structure suggested by the author of the original scale. All factors presented adequate factor loadings and semantic relations.

This study shows that, in the context of nursing, the NPIS-22-PT is a relevant instrument that can give support to the decision making of manager nurses and improve the working conditions in the organizations. The results have shown that the scale is valid and can be used in clinical practices and for investigations. This study also shows the international relevance of the use of the L’Échelle d’Interactions Infirmière-Patient-23 (EIIP-23), since it is available for few studies for the moment. Thus, it provides valuable contributions for strategic planning and health policies.

## Figures and Tables

**Table 1:** Components of the nurse-patient interaction scale 22-PT.

Items	Components		
	1 Relational Care	2 Clinical Care	3 Comfort Care
Item 9	0.699	-	-
Item 10	0.851	-	-
Item 11	0.799	-	-
Item 12	0.802	-	-
Item 13	0.831	-	-
Item 14	0.840	-	-
Item 15	0.662	-	-
Item 16	0.530	-	-
Item 17	0.608	-	-
Item 19	0.470	-	-
Item 1	-	0.651	-
Item 2	-	0.475	-
Item 3	-	0.729	-
Item 4	-	0.768	-
Item 6	-	0.825	-
Item 7	-	0.494	-
Item 8	-	0.698	-
Item 5	-	-	0.595
Item 18			0.590
Item 21	-	-	0.733
Item 22	-	-	0.688
Item 23	-	-	0.543
Explained Variance	70.183	24.433	90.219
Cronbach’s Alpha	0.910	0.827	0.745

**Table 2:** Analysis of the construct validity of EIEP-22-PT in the hospital care in Portugal.

Components	Alpha	AVE
1. Relational Care	0.910	0.52
2. Clinical Care	0.827	0.46
3. Comfort Care	0.745	0.41

## Data Availability

Restrictions apply to the availability of these data. Data was obtained from third party and are available with the permission of third party.
